# A Study on the Association Between Frailty and Primary Caregiver Burden Among Elderly Stroke Survivors

**DOI:** 10.1002/nop2.71042

**Published:** 2026-11-10

**Authors:** Sijie Wang, Huanhuan Cao, Yujie Xie, Jun Da, Li Wang, Xi Luo, Chi Zhang

**Affiliations:** ^1^ School of Nursing, Southwest Medical University Luzhou People's Republic of China; ^2^ Department of Rehabilitation Medicine The Affiliated Hospital of Southwest Medical University Luzhou People's Republic of China

**Keywords:** caregiver, caregiving burden, cross‐sectional study, frailty, stroke

## Abstract

**Aim:**

To investigate the association between multidimensional frailty among elderly stroke survivors and caregiver burden among their primary family caregivers.

**Design:**

A cross‐sectional observational study.

**Methods:**

Elderly stroke survivors and their primary family caregivers were recruited from the Department of Rehabilitation Medicine, the Affiliated Hospital of Southwest Medical University, Luzhou, Sichuan, China, between November 2025 and March 2026. Eligible stroke survivors and their primary caregivers were recruited using convenience sampling from the accessible inpatient population during the study period. Data were collected using a self‐designed general demographic questionnaire, the Tilburg Frailty Indicator (TFI) and the Caregiver Burden Inventory (CBI).

**Results:**

A total of 350 patient–caregiver pairs received questionnaires, and 323 valid paired responses were included in the analysis (effective response rate, 92.29%). The mean total Tilburg Frailty Indicator (TFI) score was 6.90 ± 4.19, and the mean total Caregiver Burden Inventory (CBI) score was 56.26 ± 16.29. Total TFI scores were positively correlated with total CBI scores (*r* = 0.647, *p* < 0.001). In the primary multivariable model, living separately from the patient and providing < 12 h of care per day (vs. > 18 h) were associated with lower caregiver burden, whereas sleeping < 6 h per day (vs. > 8 h) was associated with higher burden. The three frailty dimensions were not statistically significant after adjustment. The primary model explained 79.8% of the variance in caregiver burden (adjusted *R* ^2^ = 0.792). The expanded sensitivity model had a higher *R* ^2^ (0.962) but substantial multicollinearity (maximum VIF = 51.86), limiting the interpretability of its individual coefficients. Subgroup analyses were exploratory. The cross‐sectional design precludes causal inference.

## 1 Introduction

Stroke ranks as the leading cause of death and permanent disability among Chinese adults, with high incidence, disability, mortality and recurrence rates (Wang et al. 2019). Accelerated population aging has made frailty widespread among stroke survivors. Existing epidemiological evidence indicates that frailty is common among stroke patients, with pooled prevalence estimates varying according to the frailty definition and assessment method (Evans et al. 2025; Burton et al. 2022). In Southwest China, the rapidly aging population and increasing number of stroke survivors requiring rehabilitation have created substantial challenges for long‐term family caregiving. Elderly stroke survivors admitted to rehabilitation hospitals often present with persistent functional limitations, reduced self‐care capacity and prolonged dependence on family assistance after discharge. Although rehabilitation services primarily focus on improving patient recovery, the burden experienced by family caregivers during this transition remains insufficiently explored, particularly in relation to patients' multidimensional frailty status. Frailty has been associated with adverse outcomes after stroke, including increased mortality risk and poorer neurological and functional recovery (Burton et al. 2022; Cho et al. 2025; Wang et al. 2025; Fladt et al. 2025), and may therefore represent an important clinical marker of vulnerability among older stroke survivors (Evans et al. 2025; Burton et al. 2022; Cho et al. 2025; Wang et al. 2025; Fladt et al. 2025). Previous studies have primarily examined the effects of frailty on stroke‐related clinical outcomes, whereas the relationship between patients' multidimensional frailty and family caregiver burden remains insufficiently understood. Unlike stroke severity or disability, frailty reflects broader physiological, psychological and social vulnerability and may capture aspects of clinical vulnerability not fully represented by neurological impairment alone (Evans et al. 2025). Stroke and frailty may have a complex and potentially reciprocal relationship. Stroke‐related neurological and functional impairments may contribute to worsening frailty, while pre‐existing frailty is associated with poorer post‐stroke outcomes (Evans et al. 2025; Wang et al. 2026; Wang et al. 2025). However, frailty should be distinguished from stroke severity and disability. Stroke severity primarily reflects neurological injury, whereas disability describes limitations in daily functioning; frailty represents a broader state of vulnerability arising from accumulated deficits. Evidence in older stroke populations further suggests that frailty may provide prognostic information beyond conventional disability assessment (Forti et al. 2025). Therefore, two stroke survivors with similar neurological impairment may experience different levels of frailty and require different levels of family support. Frailty is a multidimensional construct encompassing physical, psychological and social domains (Gobbens et al. 2010). Physical frailty reflects reduced physiological reserve, including weakness, fatigue and functional decline. Psychological frailty involves emotional vulnerability and reduced psychological resilience, while social frailty represents diminished social participation, support and interpersonal resources. These interconnected dimensions may be associated with differences in patients' rehabilitation needs and the amount and type of assistance provided by family caregivers (Tyagi and Chen 2026; Rigby et al. 2009). Previous studies have extensively examined caregiver burden after stroke (Yue et al. 2022; Rigby et al. 2009; Sardella et al. 2021; Tziaka et al. 2024); however, most investigations have focused on patient disability, stroke severity or caregiver characteristics rather than the multidimensional frailty status of stroke survivors. Furthermore, evidence regarding how patient frailty interacts with caregiving circumstances in rehabilitation settings remains limited. Understanding these relationships from a patient–caregiver dyadic perspective may provide more targeted evidence for family‐centered rehabilitation planning. Rehabilitation nurses are closely involved in functional assessment, caregiver education, discharge planning and coordination of family‐centered care. Identifying the interplay between patient frailty and caregiver burden may therefore help rehabilitation nurses recognize vulnerable patient–caregiver dyads and tailor supportive care during hospitalization and discharge transitions. Therefore, this cross‐sectional study aimed to investigate the association between multidimensional frailty among elderly stroke survivors and caregiver burden among their primary family caregivers in a rehabilitation hospital in Southwest China. Specifically, this study addressed the following research questions: (1) What is the frailty profile of elderly stroke survivors undergoing rehabilitation? (2) Is patient frailty associated with caregiver burden? (3) Which patient‐ and caregiver‐related factors are statistically associated with caregiver burden after adjustment? (4) Do factors associated with caregiver burden differ across caregiver subgroups based on kinship and age? The findings may provide preliminary evidence to inform family‐centered and integrated hospital–community stroke rehabilitation services.

## 2 Subjects and Methods

### 2.1 Study Design and Setting

This was a cross‐sectional observational study conducted in the Department of Rehabilitation Medicine, the Affiliated Hospital of Southwest Medical University, Luzhou, Sichuan, China. The study aimed to investigate the association between multidimensional frailty among elderly stroke survivors and caregiver burden among their primary family caregivers. Data were collected from November 2025 to March 2026 during the patients' inpatient rehabilitation period.

### 2.2 Participants and Eligibility Criteria

Ethical approval for this study was granted by the Clinical Trial Ethics Committee of the Affiliated Hospital of Southwest Medical University on 30 October 2025 (approval No. KY2025617). Written informed consent was obtained from all participants before data collection. Participation was voluntary, and participants could withdraw from the study at any time without affecting their medical care. Inclusion criteria for stroke patients: (1) First‐ever stroke confirmed by neuroimaging and consistent with standardized stroke diagnostic criteria; (2) Aged ≥ 60 years; (3) Clinically stable with basic verbal communication capacity to complete scale assessments. Exclusion criteria for stroke patients: (1) Psychiatric disorders hindering questionnaire completion; (2) Severe multiple organ dysfunction or malignant tumours. Inclusion criteria for primary caregivers: (1) Aged ≥ 18 years; (2) an unpaid family caregiver who provided the majority of the patient's daily care or assumed primary responsibility for healthcare‐related decisions; and (3) able to communicate verbally and complete the questionnaire. When several family members shared caregiving responsibilities, the family member providing the greatest amount of daily care was identified as the primary caregiver. Exclusion criteria were: (1) simultaneously serving as the primary caregiver for another chronically ill or disabled individual; and (2) cognitive impairment or psychiatric illness that interfered with questionnaire completion. No formal effect‐size‐based a priori sample size calculation was performed before data collection. The recruitment target was determined pragmatically according to recruitment feasibility during the study period and the anticipated possibility of incomplete questionnaires. A total of 350 eligible patient–caregiver pairs received questionnaires. Of these, 27 pairs were excluded because of incomplete responses, leaving 323 complete pairs for the final analysis (effective response rate, 92.29%). The retained study records did not document the number of individuals assessed before eligibility confirmation or the number who declined participation; therefore, these counts could not be reported. The absence of prospective sample size planning should be considered when interpreting the precision and statistical power of the regression and subgroup analyses.

### 2.3 Research Instruments

#### 2.3.1 Operational Definitions

Cohabitation status referred to whether the caregiver and patient usually lived in the same household during the caregiving period. Daily caregiving duration was defined as the caregiver‐reported average number of hours per day spent directly assisting the patient. Average daily sleep duration referred to the caregiver's average self‐reported sleep time within a 24‐h period. Auxiliary caregivers referred to other family members or informal caregivers who regularly shared caregiving responsibilities with the primary caregiver. Disease course was defined as the interval between stroke onset and questionnaire assessment. Caregiver chronic conditions referred to self‐reported physician‐diagnosed chronic diseases. Smoking and alcohol status reflected the patient's self‐reported status before stroke onset.

#### 2.3.2 Self‐Designed General Demographic Questionnaire

The questionnaire collected two sets of information:

- 1: Caregiver characteristics: gender, age, educational attainment, marital status, kinship with the patient, daily care duration, average sleep duration, etc.
- 2: Patient characteristics: gender, age, cohabitation status, disease course, smoking and alcohol history, etc.

#### 2.3.3 Caregiver Burden Inventory (CBI)

The Caregiver Burden Inventory (CBI) was originally developed by Novak and Guest (1989) to assess multidimensional caregiver burden. The Chinese version was translated and validated by Yue et al. (2006). The CBI is a 24‐item self‐report scale consisting of five dimensions: time‐dependent burden, developmental burden, physical burden, social burden and emotional burden. Each item is rated on a five‐point Likert scale, with total scores ranging from 0 to 96; higher scores indicate greater caregiver burden. The Chinese version demonstrated satisfactory psychometric properties, with a Cronbach's *α* coefficient of 0.92 and a test–retest reliability coefficient of 0.93. Criterion validity was supported by a correlation of 0.85 with another established caregiver‐burden measure, and factor analysis supported the five‐dimensional structure of the instrument.

#### 2.3.4 Tilburg Frailty Indicator (TFI)

The Tilburg Frailty Indicator (TFI) was originally developed by Gobbens et al. (2010) as a multidimensional self‐report instrument for assessing physical, psychological and social frailty in older adults. The Chinese version was subsequently translated and validated by Xi et al. (2013). The TFI consists of 15 items covering three domains: physical frailty (including health‐related physical decline and functional limitations), psychological frailty (including emotional and cognitive vulnerability) and social frailty (including social relationships and support). Each item contributes to a total score ranging from 0 to 15, with higher scores indicating greater frailty. The Chinese version demonstrated acceptable psychometric properties, with a Cronbach's *α* coefficient of 0.686. Item–total correlations ranged from 0.205 to 0.620, and criterion‐related validity was supported by a significant negative correlation with the SF‐36 total score (*r* = −0.603, *p* < 0.01). The instrument also demonstrated known‐groups validity by differentiating older adults with different levels of self‐care ability.

### 2.4 Data Collection Procedures

Data collection was conducted from November 2025 to March 2026. All investigators received unified standardized training and administered questionnaires with standardized verbal instructions. For participants unable to fill out forms independently, trained researchers recorded responses through verbal interviews. All questionnaires were collected on‐site and immediately inspected for completeness, with a final valid response rate of 92.29%. Potentially eligible patients and their primary caregivers were approached as they became available during the study period. After screening according to the predefined inclusion and exclusion criteria, questionnaires were distributed to eligible pairs. Following the exclusion of incomplete paired questionnaires, 323 valid patient–caregiver pairs were included in the final analysis.

### 2.5 Statistical Analysis

Statistical analyses were performed using SPSS 26.0 software. Continuous variables were expressed as mean ± standard deviation, while categorical variables were presented as frequencies and percentages (%). Independent *t*‐tests or one‐way analysis of variance (ANOVA) were used for group comparisons. Pearson correlation analysis was performed to examine associations between frailty and caregiver burden. The primary outcome variable was caregiver burden, measured using the total CBI score. Patient frailty was examined using the total TFI score and its physical, psychological and social dimensions. Pearson correlation analysis was first performed to examine the unadjusted associations between frailty and caregiver burden. A multivariable linear regression model was subsequently constructed with the total CBI score as the dependent variable. Candidate predictors were considered based on clinical relevance, previous literature and their potential roles as confounders. Univariate statistical significance was not used as the sole criterion for variable selection. The three Tilburg Frailty Indicator (TFI) domain scores (physical, psychological and social frailty) were the primary variables of interest. The primary multivariable linear regression model retained the original model specification and included the three TFI domain scores, caregiver chronic‐condition category, patient–caregiver cohabitation status, daily caregiving duration and average daily sleep duration. This model focused on the associations between multidimensional patient frailty and caregiver burden while adjusting for key caregiving‐context characteristics.

Caregiver chronic conditions were coded as 1 = none, 2 = one, and 3 = more than one. Cohabitation status was coded as 1 = living with the patient and 2 = living separately. Daily caregiving duration was entered using dummy variables, with > 18 h/day as the reference category. Sleep duration was also entered using dummy variables, with > 8 h/day as the reference category. The three TFI domain scores were entered as continuous variables.

To assess the sensitivity of the findings to the exclusion of other clinically relevant variables that were statistically significant in univariate analyses, an expanded sensitivity model was fitted by additionally including caregiver age, caregiver educational attainment, patient age, disease course and number of auxiliary caregivers. Categorical variables in the expanded model were dummy‐coded using the following reference categories: caregiver age of 51–60 years, junior high school education, patient age of 70–79 years, disease course of < 3 months, and two to three auxiliary caregivers.

The original model was retained as the primary analysis, while the expanded model was treated as a sensitivity analysis to examine the robustness of the findings to the inclusion of additional clinically relevant variables identified in univariate analyses. The models were compared in terms of *R* ^2^, adjusted *R* ^2^, regression coefficient estimates, confidence intervals, statistical significance and variance inflation factors (VIFs). Model selection was not based on *R* ^2^ alone, because a higher *R* ^2^ does not necessarily indicate more reliable estimates of individual associations when substantial multicollinearity is present. The results of the expanded model were therefore interpreted as a sensitivity analysis rather than as definitive evidence that the expanded model was superior.

Regression diagnostics included residual plots, the Durbin–Watson statistic, and the Breusch–Pagan test for heteroscedasticity. Because heteroscedasticity was detected in both models, HC3 heteroscedasticity‐robust standard errors and corresponding inferential statistics were used to assess coefficient significance. Conventional ordinary least squares (OLS) results and HC3‐robust results were distinguished in the reporting. A two‐tailed *p* value < 0.05 was considered statistically significant.

### 2.6 Reporting Guideline

This study was reported in accordance with the Strengthening the Reporting of Observational Studies in Epidemiology (STROBE) guidelines. The completed STROBE checklist is provided as Data S1.

## 3 Results

### 3.1 Sociodemographic Characteristics of Participants

A total of 323 valid patient–caregiver pairs were included in the analysis. Among the caregivers, 60.7% (*n* = 196) were female, 56.3% (*n* = 182) were aged 51–60 years, and 57.3% (*n* = 185) had a junior high school education. Most caregivers were married with a living spouse (92.0%, *n* = 297); 52.6% (*n* = 170) were adult children of the patient, and 39.9% (*n* = 129) were spouses. Among the stroke survivors, 61.9% (*n* = 200) were male, 41.8% (*n* = 135) were aged 70–79 years, and 67.8% (*n* = 219) had a disease course of ≥ 3 months. A total of 62.2% (*n* = 201) of caregivers did not live with the patient, 32.8% (*n* = 106) reported sleeping fewer than 6 h per day, and 29.4% (*n* = 95) reported having no more than one auxiliary caregiver. The cross‐tabulation of patient sex and pre‐stroke smoking status in the original questionnaire export showed that all participants recorded as current pre‐stroke smokers were male. The variable labels, response categories and recorded values were reviewed within the available questionnaire‐platform Excel export. As completed individual questionnaires were unavailable, the observed distribution could not be independently verified and was retained as recorded in the available dataset. This finding describes the distribution in the study sample and should not be interpreted as evidence of a causal relationship between sex and smoking status. Full demographic data are summarized in Table 1.

**TABLE 1:** General demographic characteristics of participants (*n* = 323).

Variable	Category	*n*	%
Caregiver gender	Male	127	39.3
	Female	196	60.7
Caregiver education	Primary school and below	86	26.6
	Junior high school	185	57.3
	Senior high school	47	14.6
	College and above	5	1.5
Caregiver age	18–50 years	84	26.0
	51–60 years	182	56.3
	> 60 years	57	17.6
Caregiver marital status	Unmarried	3	0.9
	Married with living spouse	297	92.0
	Divorced	23	7.1
Caregiver chronic conditions	0	96	29.7
	1	125	38.7
	> 1	102	31.6
Patient gender	Male	200	61.9
	Female	123	38.1
Patient smoking history	Never smoked	115	35.6
	Former smoker	8	2.5
	Current smoker pre‐stroke	200	61.9
Patient alcohol history	Never drank alcohol	109	33.7
	Former drinker	16	5.0
	Current drinker pre‐stroke	198	61.3
Patient age	60–69 years	101	31.3
	70–79 years	135	41.8
	≥ 80 years	87	26.9
Patient disease course	< 3 months	104	32.2
	≥ 3 months	219	67.8
Cohabitation status	Live with patient	122	37.8
	Do not live with patient	201	62.2
Daily care duration	< 12 h	97	30.0
	12–18 h	125	38.7
	> 18 h	101	31.3
Average daily sleep duration	< 6 h	106	32.8
	6–8 h	89	27.6
	> 8 h	128	39.6
Number of auxiliary caregivers	0–1	95	29.4
	2–3	131	40.6
	> 3	97	30.0
Kinship with patient	Spouse	129	39.9
	Adult child	170	52.6
	Other relatives	24	7.4

### 3.2 Overall Levels of Patient Frailty and Caregiver Burden

The mean total TFI score among elderly stroke survivors was 6.90 ± 4.19. The TFI comprised three dimensions: physical, psychological, and social frailty, which were assessed using their respective scoring scales. The mean total CBI score among primary caregivers was 56.26 ± 16.29. Among the CBI dimensions, social burden had the highest mean score, whereas physical burden had the lowest mean score.

### 3.3 Correlation Between Patient Frailty and Caregiver Burden

Pearson correlation analysis confirmed a significant positive correlation between total TFI frailty score and total CBI burden score (*r* = 0.647, *p* < 0.001). Physical frailty exhibited the strongest correlation with physical care burden, and all three frailty subdimensions were positively associated with all CBI subscales (Table 2).

**TABLE 2:** Correlation coefficients between frailty subdimensions and caregiver burden subscales.

Outcome indicator	Total frailty	Physical frailty	Psychological frailty	Social frailty
Total caregiver burden	0.647**	0.561**	0.305**	0.526**
Time‐dependent burden	0.339**	0.261**	0.161**	0.361**
Developmental restriction burden	0.358**	0.296**	0.205**	0.281**
Physical burden	0.754**	0.802**	0.210**	0.414**
Social burden	0.430**	0.339**	0.244**	0.385**
Emotional burden	0.405**	0.311**	0.230**	0.379**

** *p* < 0.01, two‐tailed test.

### 3.4 Univariate Analysis of Factors Associated With Caregiver Burden

Independent‐samples *t*‐tests and one‐way ANOVA showed statistically significant differences in caregiver burden scores according to patient age, disease course, sex, pre‐stroke smoking status, alcohol history, caregiver educational attainment, caregiver age, marital status, cohabitation status, daily caregiving duration, average sleep duration and number of auxiliary caregivers (*p* < 0.05). However, the univariate associations involving patient sex and pre‐stroke smoking status are completely overlapping in the available dataset: all 200 participants recorded as male were also recorded as current pre‐stroke smokers, and all 200 participants recorded as current pre‐stroke smokers were male. Therefore, these two associations cannot be distinguished from each other or interpreted as independent associations. This observed distribution should also be interpreted cautiously because completed individual questionnaires were unavailable for independent verification. No statistically significant differences in caregiver burden were observed according to caregiver sex or caregiver chronic‐condition category (*p* > 0.05). Detailed univariate comparisons are presented in Tables 3 and 4.

**TABLE 3:** Univariate analysis of patient‐related factors associated with caregiver burden (mean ± standard deviation).

Variable	Category	*n*	Mean CBI score	Test statistic	*p*	Effect size
Patient gender	Male	200	63.81 ± 14.80	*t* = 13.14	< 0.001	Cohen's *d* = 1.51
	Female	123	43.99 ± 9.91			
Patient smoking	Never smoked	115	43.41 ± 9.94	*F* = 88.80	< 0.001	*η* ^2^ = 0.357
	Former smoker	8	52.38 ± 4.44			
	Current smoker	200	63.81 ± 14.80			
Patient alcohol	Never drank	109	43.37 ± 10.20	*F* = 86.22	< 0.001	*η* ^2^ = 0.350
	Former drinker	16	50.63 ± 6.97			
	Current drinker	198	63.82 ± 14.88			
Patient age	60–69	101	43.54 ± 10.58	*F* = 163.68	< 0.001	*η* ^2^ = 0.506
	70–79	135	54.53 ± 12.98			
	≥ 80	87	73.71 ± 9.93			
Disease course	< 3 months	104	43.43 ± 10.44	*t* = −11.61	< 0.001	Cohen's *d* = 1.38
	≥ 3 months	219	62.36 ± 14.99			

*Note:* Independent‐samples *t*‐tests were used for variables with two categories, for which Cohen's *d* was reported as the effect size. One‐way analysis of variance (ANOVA) was used for variables with three or more categories, for which eta squared (*η* ^2^) was reported. All 200 participants recorded as male were also recorded as current pre‐stroke smokers; therefore, the associations of patient sex and pre‐stroke smoking status cannot be distinguished in this dataset. Completed individual questionnaires were unavailable for independent verification.

Abbreviation: CBI, Caregiver Burden Inventory.

**TABLE 4:** Univariate analysis of the impact of primary caregivers with different characteristics on caregiver burden (mean ± standard deviation) unit: points.

Variable	Category	*n*	Mean CBI score	Test statistic	*p*	Effect size
Caregiver gender	Male	127	55.68 ± 16.30	*t =* −0.52	0.60	Cohen's *d* = 0.06
	Female	196	56.64 ± 16.32			
Education	Primary and below	86	78.85 ± 5.80	*F* = 514.07	< 0.001	*η* ^2^ = 0.829
	Junior high	185	51.61 ± 7.88			
	Senior high	47	36.49 ± 2.73			
	College+	5	26.00 ± 2.35			
Caregiver age	18–50	84	37.89 ± 4.52	*F* = 583.94	< 0.001	
	51–60	182	56.58 ± 9.39			*η* ^2^ = 0.785
	> 60	57	82.33 ± 3.60			
Marital status	Unmarried	3	24.33 ± 0.58	*F* = 64.73	< 0.001	*η* ^2^ = 0.288
	Married	297	54.28 ± 14.33			
	Divorced	23	86.04 ± 2.06			
Caregiver chronic disease	0	96	57.74 ± 17.82	*F* = 0.83	0.44	*η* ^2^ = 0.005
	1	125	54.92 ± 15.32			
	> 1	102	56.52 ± 15.98			
Cohabitation	Live together	122	74.59 ± 8.41	*t* = 32.80	< 0.001	Cohen's *d* = 3.76
	Separate residence	201	45.14 ± 7.45			
Daily care hours	< 12 h	97	42.68 ± 8.99	*F* = 158.76	< 0.001	*η* ^2^ = 0.498
	12–18 h	125	54.30 ± 13.20			
	> 18 h	101	71.74 ± 11.63			
Daily sleep	< 6 h	106	60.58 ± 17.93	*F* = 6.85	0.001	*η* ^2^ = 0.041
	6–8 h	89	56.11 ± 15.33			
	> 8 h	128	52.80 ± 14.73			
Auxiliary caregivers	0–1	95	71.83 ± 11.59	*F* = 155.69	< 0.001	*η* ^2^ = 0.493
	2–3	131	55.34 ± 13.67			
	> 3	97	42.26 ± 8.18			
Kinship	Spouse	129	56.22 ± 15.71	*F* = 0.72	0.49	
	Child	170	56.82 ± 17.20			*η* ^2^ = 0.004
	Other relatives	24	52.54 ± 12.34			

*Note:* Independent‐samples *t*‐tests were used for variables with two categories, for which Cohen's d was reported as the effect size. One‐way analysis of variance (ANOVA) was used for variables with three or more categories, for which eta squared (*η* ^2^) was reported.

Abbreviation: CBI, Caregiver Burden Inventory.

### 3.5 Multiple Linear Regression of Caregiver Burden

Following assessment of clinical relevance and multicollinearity among candidate variables, the primary multivariable linear regression model included the three TFI frailty dimensions, caregiver chronic conditions, patient–caregiver cohabitation status, daily caregiving duration and sleep duration. The conventional OLS model was statistically significant (*F* (9, 313) = 137.096, *p* < 0.001), explaining 79.8% of the variance in caregiver burden (*R* ^2^ = 0.798; adjusted *R* ^2^ = 0.792). The HC3‐robust Wald test also indicated that the model was statistically significant (*F* (9, 313) = 132.29, *p* < 0.001). The coding rules for the variables included in the primary multivariable regression model are summarized in Table 5.

**TABLE 5:** Coding of variables included in the primary multivariable linear regression model.

Variable	Coding rules
Caregiver chronic conditions	1 = none; 2 = one; 3 = more than one
Cohabitation status	1 = live with patient; 2 = separate residence
Daily caregiving duration: < 12 h	1 = yes; 0 = no
Daily caregiving duration: 12–18 h	1 = yes; 0 = no
Daily caregiving duration: > 18 h	Reference category
Sleep duration: < 6 h	1 = yes; 0 = no
Sleep duration: 6–8 h	1 = yes; 0 = no
Sleep duration: > 8 h	Reference category
Physical frailty	Continuous score
Psychological frailty	Continuous score
Social frailty	Continuous score

*Note:* Daily caregiving duration and sleep duration were entered as dummy variables, with > 18 h/day and > 8 h/day as the respective reference categories. Caregiver chronic conditions were entered as an ordinal variable coded as 1 = none, 2 = one, and 3 = more than one. Physical, psychological and social frailty scores were entered as continuous variables.

HC3‐robust inference showed that living separately from the patient was associated with lower caregiver burden compared with living with the patient (*B* = −24.506, *p* < 0.001). Providing < 12 h of care per day, compared with > 18 h, was associated with lower caregiver burden (*B* = −5.500, *p* = 0.005). Sleeping fewer than 6 h per day, compared with > 8 h, was associated with higher caregiver burden (*B* = 2.855, *p* = 0.039). The physical, psychological, and social frailty dimensions were not statistically significant in the adjusted model. The full results of the primary multivariable regression model are presented in Table 6. The corresponding HC3‐robust coefficient estimates for the primary multivariable regression model are also provided in Supplementary Table S1.

**TABLE 6:** Primary multivariable linear regression analysis of factors associated with caregiver burden.

Predictor	*B*	HC3 robust SE	95% CI for *B*	*β*	*t*	*p*
Constant	93.298	4.027	85.375 to 101.221	—	23.17	< 0.001
Caregiver chronic conditions	0.844	1.088	−1.297 to 2.986	0.04	0.78	0.438
Cohabitation status (separate residence vs. living with patient)	−24.506	1.372	−27.205 to −21.807	−0.73	−17.87	< 0.001
Daily care < 12 h (vs. > 18 h)	−5.500	1.963	−9.361 to −1.638	−0.16	−2.80	0.005
Daily care 12–18 h (vs. > 18 h)	−1.187	1.526	−4.190 to 1.816	−0.04	−0.78	0.437
Sleep < 6 h (vs. > 8 h)	2.855	1.376	0.147 to 5.563	0.08	2.07	0.039
Sleep 6–8 h (vs. > 8 h)	0.294	1.438	−2.535 to 3.123	0.01	0.20	0.838
Physical frailty	0.500	0.318	−0.126 to 1.127	0.06	1.57	0.117
Psychological frailty	0.115	0.236	−0.349 to 0.579	0.01	0.49	0.626
Social frailty	0.309	0.278	−0.239 to 0.857	0.04	1.11	0.268

*Note:* *N* = 323. *R* ^2^ = 0.798 and adjusted *R* ^2^ = 0.792. The conventional OLS model was statistically significant (*F* (9, 313) = 137.096, *p* < 0.001); the HC3‐robust Wald test was also statistically significant (*F* (9, 313) = 132.29, *p* < 0.001). HC3 heteroscedasticity‐robust standard errors, 95% confidence intervals, *t* statistics, and *p* values are reported. The Breusch–Pagan test indicated heteroscedasticity (*p* = 0.0000066). All VIF values in the primary model were below 5 (maximum VIF = 4.99). The reference categories were > 18 h/day for daily caregiving duration and > 8 h/day for sleep duration. Caregiver chronic conditions were coded as 1 = none, 2 = one, and 3 = more than one. Cohabitation status was coded as 1 = living with the patient and 2 = living separately.

Abbreviations: *β*, standardized regression coefficient; *B*, unstandardized regression coefficient; CI, confidence interval; HC3, heteroscedasticity‐consistent covariance estimator; SE, standard error.

An expanded sensitivity model was fitted by additionally including caregiver age, caregiver educational attainment, patient age, disease course and number of auxiliary caregivers. Compared with the primary model, *R* ^2^ increased from 0.798 to 0.962 and adjusted *R* ^2^ increased from 0.792 to 0.960. However, the maximum variance inflation factor (VIF) increased from 4.99 to 51.86, indicating substantial multicollinearity in the expanded model.

HC3‐robust inference showed that caregiver age and educational attainment remained statistically associated with caregiver burden in the expanded model, whereas the patient‐age categories, disease course and auxiliary‐caregiver categories were not statistically significant. Several of these latter predictors had high VIF values, limiting the interpretation of their individual adjusted coefficients. The coefficient for separate residence was attenuated from −24.506 in the primary model to −12.985 in the expanded model. For caregivers providing < 12 h of care per day, the expanded‐model conventional OLS *p* value was 0.242, whereas the HC3‐robust *p* value was 0.027. Sleep duration of < 6 h was not statistically significant in the expanded model using HC3‐robust inference.

Because heteroscedasticity was detected in both models, HC3‐robust standard errors were used for coefficient‐level confidence intervals and *p* values in the model comparison. Conventional OLS model‐fit statistics are reported separately. Given the substantial multicollinearity and changes in coefficient estimates, the expanded model was retained as a sensitivity analysis rather than the primary model. The findings should be interpreted as model‐dependent associations rather than definitive independent or causal effects. Detailed comparisons are provided in Table 7.

**TABLE 7:** Comparison of the primary and expanded sensitivity regression models.

Predictor	Model 1: *B* (95% CI)	HC3 *p*	Model 2: *B* (95% CI)	HC3 *p*	Model 2 VIF
Caregiver chronic conditions	0.844 (−1.297, 2.986)	0.438	0.130 (−0.953, 1.214)	0.813	5.24
Separate residence	−24.506 (−27.205, −21.807)	< 0.001	−12.985 (−14.414, −11.556)	< 0.001	3.67
Daily care < 12 h	−5.500 (−9.361, −1.638)	0.005	−2.290 (−4.314, −0.266)	0.027	24.21
Daily care 12–18 h	−1.187 (−4.190, 1.816)	0.437	0.725 (−1.594, 3.044)	0.539	8.80
Sleep < 6 h	2.855 (0.147, 5.563)	0.039	−0.299 (−1.705, 1.108)	0.676	2.77
Sleep 6–8 h	0.294 (−2.535, 3.123)	0.838	−0.142 (−1.490, 1.205)	0.836	2.91
Physical frailty	0.500 (−0.126, 1.127)	0.117	0.119 (−0.199, 0.437)	0.462	2.76
Psychological frailty	0.115 (−0.349, 0.579)	0.626	−0.112 (−0.316, 0.092)	0.282	1.32
Social frailty	0.309 (−0.239, 0.857)	0.268	0.068 (−0.207, 0.343)	0.626	1.71
Caregiver age 18–50 vs. 51–60	—	—	−8.482 (−9.382, −7.581)	< 0.001	2.65
Caregiver age > 60 vs. 51–60	—	—	10.283 (9.029, 11.536)	< 0.001	2.59
Primary/below vs. junior high	—	—	7.353 (5.970, 8.735)	< 0.001	4.28
Senior high vs. junior high	—	—	−4.918 (−5.917, −3.919)	< 0.001	2.46
College+ vs. junior high	—	—	−14.985 (−17.389, −12.581)	< 0.001	1.23
Patient age 60–69 vs. 70–79	—	—	2.242 (−0.991, 5.475)	0.173	51.86
Patient age ≥ 80 vs. 70–79	—	—	2.065 (−0.695, 4.826)	0.142	10.56
Disease course ≥ 3 months	—	—	−0.363 (−2.922, 2.196)	0.780	26.12
Auxiliary caregivers 0–1 vs. 2–3	—	—	−0.145 (−2.809, 2.519)	0.915	12.42
Auxiliary caregivers > 3 vs. 2–3	—	—	−0.611 (−3.694, 2.473)	0.697	26.72

*Note:* *N* = 323 in both models. Model 1: 9 predictor terms; *R* ^2^ = 0.798; adjusted *R* ^2^ = 0.792; AIC = 2222.42; conventional OLS *F* (9, 313) = 137.096, *p* < 0.001; maximum VIF = 4.99. Model 2: 19 predictor terms; *R* ^2^ = 0.962; adjusted *R* ^2^ = 0.960; AIC = 1701.37; conventional OLS *F* (19, 303) = 404.850, *p* < 0.001; maximum VIF = 51.86. Breusch–Pagan tests indicated heteroscedasticity in both models (Model 1 *p* = 0.00000659; Model 2 *p* = 0.00000165). Coefficients, 95% CIs and *p* values use HC3 robust standard errors; model‐fit statistics and overall *F* tests are conventional OLS statistics. Reference categories: caregiver age 51–60 years; junior high school education; patient age 70–79 years; disease course < 3 months; 2–3 auxiliary caregivers; > 18 h/day caregiving; and > 8 h/day sleep.

Abbreviations: *B*, unstandardized regression coefficient; CI, confidence interval; HC3, heteroscedasticity‐consistent covariance estimator; VIF, variance inflation factor.

### 3.6 Exploratory Stratified Analyses Across Caregiver Subgroups

Stratified multiple linear regression analyses were conducted as exploratory analyses according to caregiver kinship and age. The ‘other relatives’ subgroup was not included in regression analysis because of its limited sample size. The models for spousal and adult‐child caregivers were statistically significant overall. Among spousal caregivers, cohabitation status, daily caregiving duration and sleep duration were associated with caregiver burden, whereas the frailty dimensions were not statistically associated with burden after adjustment. Among adult‐child caregivers, cohabitation status remained significantly associated with caregiver burden; however, multicollinearity involving the sleep‐duration dummy variables limited interpretation of this model. Age‐stratified analyses suggested possible associations of psychological and social frailty with caregiver burden in some younger and middle‐aged caregiver subgroups, whereas the model for caregivers aged > 60 years was not statistically significant. Given the relatively small sample sizes within several strata, these subgroup findings should be regarded as exploratory and interpreted cautiously.

## 4 Discussion

This study provides evidence that caregiver burden among primary family caregivers of elderly stroke survivors is associated with both patient vulnerability and caregiving context. Although patient frailty demonstrated a strong positive correlation with caregiver burden, frailty dimensions were no longer statistically significant after adjustment for caregiving‐related characteristics. Instead, cohabitation status, daily caregiving duration and sleep duration remained statistically associated with caregiver burden in the adjusted model. These findings suggest that frailty may represent an important marker of caregiving complexity, but caregiver burden is also strongly associated with the social and behavioural context in which caregiving occurs.

### 4.1 Frailty Profiles Among Hospitalized Elderly Stroke Survivors

This study assessed physical, psychological, and social frailty among hospitalized elderly stroke survivors. Previous evidence indicates that frailty is common among stroke survivors and is associated with impaired recovery trajectories and adverse clinical outcomes (Burton et al. 2022; Cho et al. 2025; Wang et al. 2025; Fladt et al. 2025).

Physical frailty among elderly stroke survivors may reflect the interaction between stroke‐related neurological impairment and age‐related physiological decline. Unlike stroke severity, which primarily captures neurological damage, frailty reflects reduced physiological reserve and diminished capacity to recover from stressors. Therefore, elderly stroke survivors with similar neurological deficits may experience different rehabilitation trajectories depending on their underlying frailty status.

From a rehabilitation perspective, these findings suggest that assessment limited to motor impairment or functional disability may underestimate the complexity of recovery needs. Incorporating multidimensional frailty assessment, including physical, psychological and social domains, may help clinicians identify patients requiring additional family and community support after discharge.

These findings support the clinical value of incorporating multidimensional frailty assessment into rehabilitation evaluation, while prospective studies are needed to determine whether targeted frailty interventions improve patient and caregiver outcomes.

### 4.2 Association Between Patient Frailty and Caregiver Burden

Consistent with previous research, greater care‐recipient frailty has been associated with greater caregiver burden (Tyagi and Chen 2026; Ringer et al. 2017; Sardella et al. 2021), which is consistent with the positive correlation observed in the present study. In elderly stroke survivors, this association may reflect the combination of stroke‐related functional limitations and age‐related reductions in physiological reserve. Greater physical frailty may coincide with increased assistance needs for mobility, personal care, feeding and rehabilitation exercises, thereby increasing the amount of time and effort required from family caregivers. Psychological and social frailty may represent additional sources of caregiving complexity because emotional vulnerability, reduced social participation, communication difficulties and limited patient engagement may increase caregivers' supportive responsibilities (Tziaka et al. 2024; Haley et al. 2015).

Importantly, total TFI score was positively correlated with caregiver burden in the unadjusted correlation analysis. However, the primary multivariable model included the physical, psychological and social frailty domain scores rather than the total TFI score. None of the three frailty domain scores was statistically significant after adjustment. Therefore, the adjusted findings should be interpreted as evidence that the domain‐specific associations were not statistically significant in the primary model, rather than as a direct estimate of the adjusted association between total frailty and caregiver burden. This finding suggests that the association between patient frailty and caregiver burden should be interpreted in the context of caregiving circumstances and other potentially relevant characteristics. However, the absence of statistically significant adjusted coefficients does not establish that patient frailty has no relationship with caregiver burden.

The expanded sensitivity analysis further highlighted the importance of model specification. Caregiver age and educational attainment remained statistically associated with caregiver burden in the expanded model, and their VIF values did not indicate severe multicollinearity. Their exclusion from the primary model should therefore not be interpreted as evidence that these variables were unimportant. Rather, retaining the original model specification meant that potential residual confounding by caregiver characteristics could not be ruled out. Patient age, disease course and number of auxiliary caregivers were also examined in the expanded model, but substantial multicollinearity among several predictors limited the interpretation of their individual adjusted coefficients. Although the expanded model had a higher *R* ^2^, this alone does not establish that it provides more reliable estimates of individual associations. Changes in coefficient estimates and statistical significance indicate that the findings were sensitive to model specification.

Taken together, these findings suggest that caregiver burden may reflect the combined influence of patient vulnerability and the circumstances in which care is provided, including cohabitation, caregiving intensity and opportunities for caregiver recovery. Because this was a cross‐sectional study, the observed associations cannot establish causal relationships. The findings should be interpreted as model‐dependent associations, and the possibility of residual confounding should be acknowledged. Future longitudinal studies with prospectively planned analyses and more comprehensive assessment of patient clinical characteristics and caregiving circumstances are needed to clarify the relationship between patient frailty and caregiver burden.

### 4.3 Factors Associated With Caregiver Burden

In the present study, patient cohabitation status showed the largest regression coefficient among the variables retained in the adjusted model. Caregivers living with stroke survivors reported higher caregiver burden scores compared with those living separately. Continuous co‐residence may increase exposure to daily caregiving demands, reduce opportunities for psychological recovery and limit caregivers' ability to maintain personal routines. Previous studies have demonstrated that greater caregiving involvement and prolonged daily exposure to caregiving tasks are associated with greater caregiver burden among stroke survivors and other older adults requiring long‐term assistance (Yue et al. 2022; Tooth et al. 2005). Therefore, rehabilitation programs should pay particular attention to families providing continuous co‐residential care and consider incorporating respite care and community‐based support services. Daily caregiving duration was another significant factor associated with caregiver burden. In this study, caregivers providing < 12 h of daily care reported lower burden than those providing longer caregiving hours. This finding supports previous evidence that increased time commitment and caregiving intensity are closely related to physical exhaustion, emotional stress and reduced quality of life among family caregivers (Tziaka et al. 2024; Tooth et al. 2005; Tsiakiri et al. 2023; Yu et al. 2013). Greater caregiving intensity may be accompanied by reduced opportunities for rest and self‐care, which have been associated with poorer caregiver well‐being in previous studies. Sleep duration was also independently associated with caregiver burden in the adjusted model. Caregivers sleeping fewer than 6 h per day experienced higher burden, suggesting that insufficient recovery time may exacerbate caregiving‐related stress. Previous studies have emphasized that caregiver burden is closely linked with psychological distress, impaired health status and reduced quality of life among stroke caregivers (Yue et al. 2022; Tooth et al. 2005; Liu et al. 2020; Tsiakiri et al. 2023). In the present study, shorter sleep duration may reflect limited opportunities for physical and psychological recovery; however, because sleep was assessed cross‐sectionally, it should be interpreted as an associated caregiving characteristic rather than a causal pathway. Although patient frailty demonstrated significant correlations with caregiver burden in Pearson correlation analyses, frailty dimensions did not remain statistically significant after adjustment in the multivariable model. The attenuation of the frailty coefficients after adjustment may indicate shared variance between frailty‐related care needs and caregiving‐context variables; however, the cross‐sectional design and the absence of several clinical covariates preclude a definitive explanation. Previous studies have reported associations between patient frailty and caregiver burden, potentially reflecting greater care dependency and functional demands among frail care recipients (Tyagi and Chen 2026; Sardella et al. 2021). Future longitudinal studies incorporating stroke severity, functional disability, cognitive status and social support variables are needed to clarify the mechanisms underlying the relationship between frailty progression and caregiver burden. Taken together, these findings indicate that caregiver burden is associated with both patient‐related and caregiving‐context characteristics. Support strategies for caregivers with greater caregiving demands should therefore combine patient rehabilitation with individualized caregiver support.

### 4.4 Heterogeneous Burden Patterns Across Caregiver Subgroups

The exploratory subgroup analyses suggested that patterns of factors associated with caregiver burden may differ according to caregiver kinship and age. Among spousal caregivers, cohabitation, caregiving duration and sleep duration were associated with burden, whereas cohabitation was the principal factor retained in the adult‐child model. Age‐stratified analyses also suggested possible differences in the associations of psychological and social frailty with burden. These findings may reflect differences in caregiving roles, available resources and daily caregiving demands across family members, as reported in previous stroke‐caregiving research (Tooth et al. 2005; Blom Johansson et al. 2022).

However, these subgroup findings should be interpreted cautiously. The number of participants in several subgroups was limited; the ‘other relatives’ group could not be modelled; multicollinearity affected part of the adult‐child analysis; and the model for caregivers aged > 60 years was not statistically significant. Therefore, these analyses should be considered hypothesis‐generating rather than confirmatory. Larger multicentre studies are needed before subgroup‐specific associations can be used to guide targeted interventions.

### 4.5 Study Limitations

This study has several limitations. First, participants were recruited using convenience sampling from a single rehabilitation hospital, which may limit the generalizability of the findings to other rehabilitation settings, acute stroke populations and community‐dwelling stroke survivors. Second, the cross‐sectional design permits identification of associations but cannot establish temporal or causal relationships between patient frailty and caregiver burden. Third, several subgroup analyses were based on relatively small samples, and multicollinearity affected some subgroup models; therefore, these exploratory results may be statistically unstable. Fourth, several clinically important stroke‐related characteristics, including stroke severity, functional independence, cognitive status, depressive symptoms, communication impairment and detailed comorbidity burden, were not collected in the original study protocol. Their omission may have resulted in residual confounding because these characteristics may be associated with both patient frailty and caregiving demands. Finally, frailty and caregiver burden were assessed using questionnaire‐based measures, which may be susceptible to recall and reporting bias.

An additional limitation concerns the observed overlap between patient sex and pre‐stroke smoking status. In the available questionnaire‐platform Excel export, all 200 participants recorded as male were also recorded as current pre‐stroke smokers, and all 200 participants recorded as current pre‐stroke smokers were male. Although the recorded labels and coding categories were checked within the available export, completed individual questionnaires were unavailable for independent verification. Therefore, the accuracy of this distribution could not be independently confirmed, and the separate associations of sex and smoking status should be interpreted cautiously. In addition, no formal effect‐size‐based a priori sample‐size calculation was performed before data collection. Therefore, the precision of the estimates and the ability to detect smaller effects, particularly in subgroup analyses, may have been limited. Future studies should incorporate prospective sample‐size planning based on prespecified primary outcomes and clinically meaningful effect sizes. Multicentre longitudinal studies incorporating more comprehensive clinical assessments are needed to confirm these associations and clarify their temporal relationships. The primary model did not include caregiver age and educational attainment, both of which remained statistically associated with caregiver burden in the expanded sensitivity model. Their omission may have resulted in residual confounding. In addition, substantial multicollinearity among patient age, disease course, caregiving duration and auxiliary caregiver availability limited the interpretability of their simultaneous adjusted estimates. The regression findings should therefore be interpreted cautiously as model‐dependent associations rather than definitive independent effects.

## 5 Clinical Implications

The findings have several implications for rehabilitation nursing and family‐centered stroke care. Rehabilitation nurses are well positioned to assess both patient frailty and caregiver burden during inpatient rehabilitation and discharge planning. Particular attention may be warranted for caregivers who co‐reside with the patient, provide prolonged daily care or report insufficient sleep because these characteristics were statistically associated with greater caregiver burden in the adjusted model. Nursing assessment may therefore incorporate the TFI for frailty screening and the CBI for multidimensional assessment of caregiver burden. For caregivers reporting greater burden or limited recovery time, rehabilitation teams may consider individualized education, respite resources, sleep and self‐care support and referral to community services. Coordination between hospital rehabilitation teams and community health services may further support continuity of patient rehabilitation and caregiver assistance after discharge. Because the present study was cross‐sectional, the effectiveness of these strategies should be evaluated in prospective intervention studies.

## 6 Conclusion

This cross‐sectional study found that caregiver burden among family caregivers of elderly stroke survivors was statistically associated with patient–caregiver cohabitation status, daily caregiving duration and sleep duration. Although the total TFI score was positively correlated with caregiver burden, none of the three frailty domain scores was statistically significant in the primary adjusted model. These findings highlight the importance of considering both patient frailty and caregiving circumstances when assessing caregiver burden and integrating frailty assessment with caregiver support during rehabilitation. Future multicentre longitudinal studies are needed to clarify temporal relationships and determine whether interventions targeting patient and caregiver needs can reduce caregiver burden.

## Author Contributions

**Sijie Wang:** conceptualization, investigation, validation, methodology, data curation. **Huanhuan Cao:** writing – review and editing, data curation, investigation, conceptualization, writing – original draft. **Yujie Xie:** conceptualization, investigation, validation, software, writing – original draft. **Jun Da:** conceptualization, investigation, writing – original draft. **Li Wang:** conceptualization, investigation, writing – original draft, formal analysis. **Xi Luo:** conceptualization, investigation, writing – original draft, data curation, validation, methodology. **Chi Zhang:** conceptualization, investigation, funding acquisition, writing – original draft, writing – review and editing, methodology.

## Funding

This work was supported by the Sichuan Provincial Department of Science and Technology (Grant No. 2024YFHZ0050); Luzhou Science and Technology Bureau (Grant Nos. 2024LZXNYDJ035, 2020LZXNYDJ14); Cooperative Research Project of Deyang Second People's Hospital and Southwest Medical University (Grant No. 2022DYEXNYD002); and the Special Project of Central Government Guiding Local Science and Technology Development of Sichuan Province (Grant No. 2024ZYD0269).

## Ethics Statement

Ethical approval for this study was granted by the Clinical Trial Ethics Committee of the Affiliated Hospital of Southwest Medical University on 30 October 2025 (approval No. KY2025617). Written informed consent was obtained from all participants before data collection.

## Conflicts of Interest

The authors declare no conflicts of interest.

## Supporting information


**Data S1:** Supporting Information.

**Supplementary Table S1:** HC3 Heteroscedasticity‐Robust Regression Results for the Primary Multivariable Linear Regression Model.

## Data Availability

The data that support the findings of this study are available on request from the corresponding author. The data are not publicly available due to privacy or ethical restrictions.
