# Supplementary material for: A Study on the Association Between Frailty and Primary Caregiver Burden Among Elderly Stroke Survivors
Source: Nurs Open. 2026 Nov 10;13(11):e71042. doi: 10.1002/nop2.71042 (PMC13751824; doi:10.1002/nop2.71042)
Supplement: Supplementary file 1 [file NOP2-13-e71042-s001.docx]

Supplementary Table S1. Supplementary Table S1. HC3 Heteroscedasticity-Robust Regression Results for the Primary Multivariable Linear Regression Model

| Variable | B | HC3 robust SE | 95% CI for B | P |
| --- | --- | --- | --- | --- |
| Constant | 93.298 | 4.027 | 85.375 to 101.221 | <0.001 |
| Caregiver chronic conditions | 0.844 | 1.088 | -1.297 to 2.986 | 0.438 |
| Separate residence | -24.506 | 1.372 | -27.205 to -21.807 | <0.001 |
| Daily care <12 h | -5.500 | 1.963 | -9.361 to -1.638 | 0.005 |
| Daily care 12–18 h | -1.187 | 1.526 | -4.190 to 1.816 | 0.437 |
| Sleep <6 h | 2.855 | 1.376 | 0.147 to 5.563 | 0.039 |
| Sleep 6–8 h | 0.294 | 1.438 | -2.535 to 3.123 | 0.838 |
| Physical frailty | 0.500 | 0.318 | -0.126 to 1.127 | 0.117 |
| Psychological frailty | 0.115 | 0.236 | -0.349 to 0.579 | 0.626 |
| Social frailty | 0.309 | 0.278 | -0.239 to 0.857 | 0.268 |

Note. B = unstandardized regression coefficient; HC3 robust SE = heteroscedasticity-consistent standard error (HC3); CI = confidence interval. The 95% CIs and P values use the HC3 covariance estimator and residual degrees of freedom. Reference groups: living with the patient, >18 h/day of care, and >8 h/day of sleep. N = 323. Model R² = 0.798; adjusted R² = 0.792.
