# Supplementary material for: A Study on the Association Between Frailty and Primary Caregiver Burden Among Elderly Stroke Survivors
Source: Nurs Open. 2026 Nov 10;13(11):e71042. doi: 10.1002/nop2.71042 (PMC13751824; doi:10.1002/nop2.71042)
Supplement: Supplementary file 2 [file NOP2-13-e71042-s002.docx]

**STROBE Checklist — Cross-Sectional Study**

*Manuscript: A Study on the Association Between Frailty and Primary Caregiver Burden Among Elderly Stroke Survivors*

Checklist locations are mapped to the uploaded blinded manuscript (41 pages). Items described as not applicable or not separately reported are identified transparently

| **STROBE item** | **Recommendation** | **Location in manuscript** |
| --- | --- | --- |
| **1a Title and abstract** | Identify the study design in the title or abstract. | Abstract, p. 1 (the abstract identifies the study as cross-sectional; the title does not name the design). |
| **1b Abstract** | Provide an informative and balanced summary. | Abstract, p. 1. |
| **2 Background/rationale** | Explain the scientific background and rationale. | Introduction, pp. 2–5. |
| **3 Objectives** | State specific objectives and any prespecified hypotheses. | Introduction, pp. 4–5 (objectives and research questions are stated; no prespecified hypothesis is explicitly reported). |
| **4 Study design** | Present the key design elements early in the paper. | Section 2.1, p. 5. |
| **5 Setting** | Describe the setting, location, and relevant dates. | Sections 2.1 and 2.4, pp. 5 and 9 (rehabilitation department of a tertiary rehabilitation hospital in Southwest China; November 2025–March 2026). |
| **6a Participants** | Give eligibility criteria and participant selection methods. | Sections 2.2 and 2.4, pp. 6–7 and 9. Section 2.2 describes the inclusion and exclusion criteria for stroke survivors and primary caregivers. Section 2.4 describes convenience sampling from the accessible inpatient population, with potentially eligible patient–caregiver pairs approached as they became available during the study period. |
| **6b Matched studies** | For matched studies, give matching criteria and numbers. | Not applicable: this is a cross-sectional study without a matched design. |
| **7 Variables** | Define outcomes, exposures, predictors, confounders, and effect modifiers. | Sections 2.3.1–2.3.2 and 2.5, pp. 7–11. |
| **8 Data sources/measurement** | Describe data sources and measurement methods. | Sections 2.3.2–2.3.4, pp. 7–9 (demographic questionnaire, CBI, and TFI). |
| **9 Bias** | Describe steps taken to address potential sources of bias. | Section 2.4, p. 9 (standardized training/instructions and completeness checks); Section 4.5, pp. 34–36 (reporting/recall bias limitation). |
| **10 Study size** | Explain how the study size was determined. | Section 2.2, p. 6; Section 4.5, pp. 34–36 (no formal a priori effect-size/power calculation; pragmatic recruitment target). |
| **11 Quantitative variables** | Explain how quantitative variables were handled and why category boundaries were chosen. | Sections 2.3.1 and 2.5, pp. 7 and 10–11; Tables 1 and 3–5, pp. 12–20 (category definitions and reference groups are shown; the rationale for selecting cut-points is not fully detailed). |
| **12a Statistical methods** | Describe statistical methods, including methods used to control confounding. | Section 2.5, pp. 10–11 (primary and expanded sensitivity models, model comparison, VIF, regression diagnostics, and HC3-robust inference). |
| **12b Subgroups/interactions** | Describe methods used for subgroup or interaction analyses. | Sections 2.5 and 3.6, pp. 10–11 and 27 (exploratory stratified analyses; no interaction analysis is explicitly reported). |
| **12c Missing data** | Explain how missing data were handled. | Section 2.2, p. 6 (27 incomplete patient–caregiver pairs were excluded; 323 complete pairs were analyzed). |
| **12d Loss to follow-up** | For longitudinal studies, explain loss to follow-up. | Not applicable: this is a cross-sectional study. |
| **12e Sensitivity analyses** | Describe sensitivity analyses. | Section 2.5, pp. 10–11; Section 3.5, pp. 20–26 (the expanded sensitivity model is compared with the primary model in Table 7, pp. 25–26). |
| **13a Participant numbers** | Report numbers of individuals at each study stage, including those included and analyzed. | Section 2.2, p. 6; Section 3.1, p. 12 (350 patient–caregiver pairs received questionnaires; 27 incomplete pairs excluded; 323 analyzed). |
| **13b Non-participation** | Give reasons for non-participation at each stage, if available. | Section 2.2, p. 6 (27 incomplete pairs excluded; screening counts and numbers declining participation were not documented in retained records). |
| **13c Flow diagram** | Consider using a participant flow diagram. | No flow diagram is included in the current manuscript; participant counts are described in Section 2.2, p. 6. |
| **14a Descriptive data** | Give participant characteristics and relevant clinical/social information. | Section 3.1 and Table 1, pp. 12–15. |
| **14b Missing data by variable** | Report the number of participants with missing data for each variable. | No missing values were recorded for the variables included in the analyses among the 323 included patient–caregiver pairs. |
| **14c Follow-up data** | For cohort studies, summarize follow-up time. | Not applicable: this is a cross-sectional study. |
| **15 Outcome data** | Report outcome data or summary measures. | Section 3.2, pp. 15–16; Sections 3.2–3.5, pp. 15–26 (TFI and CBI summary scores, correlation and univariate results, and primary and sensitivity regression outcomes). |
| **16a Main results** | Give unadjusted and adjusted estimates with precision and identify adjustment variables. | Tables 2–7, pp. 16–26; Section 3.5, pp. 20–26 (Table 6 reports HC3-robust estimates for the primary model, and Table 7 compares the primary and expanded sensitivity models using HC3-robust coefficient estimates, 95% confidence intervals, P values, and VIFs); adjustment variables and statistical methods are described in Section 2.5, pp. 10–11. |
| **16b Category boundaries** | Report boundaries for categorized quantitative variables. | Sections 2.3.1 and 2.5, pp. 7 and 10–11; Tables 1 and 3–5, pp. 12–20. |
| **16c Absolute risk translation** | Translate relative risks into absolute risks when relevant. | Not applicable: the outcome is continuous caregiver-burden score analyzed using linear regression. |
| **17 Other analyses** | Report other analyses, including subgroup and sensitivity analyses. | Section 3.5, pp. 20–26 (primary and expanded sensitivity regression models); Section 3.6, p. 27 (exploratory stratified analyses across caregiver subgroups). |
| **18 Key results** | Summarize key results in relation to the study objectives. | Discussion, pp. 27–36; Conclusion, pp. 36–37. |
| **19 Limitations** | Discuss limitations, including bias and imprecision. | Section 4.5, pp. 34–36 (single-centre recruitment, cross-sectional design, small subgroups, omitted clinical confounders, reporting/recall bias, the overlap between patient sex and pre-stroke smoking status, and no formal a priori sample-size calculation). |
| **20 Interpretation** | Give a cautious interpretation considering objectives, limitations, multiplicity, and evidence. | Discussion, Sections 4.1–4.5, pp. 28–36 (associations are distinguished from causal effects; subgroup findings are described as exploratory, and the sensitivity-model findings are interpreted cautiously in view of multicollinearity and possible residual confounding). |
| **21 Generalisability** | Discuss the external validity/generalizability of the findings. | Section 4.5, pp. 34–36. |
| **22 Funding** | Report funding sources and the role of funders. | Sichuan Provincial Department of Science and Technology — Grant No. 2024YFHZ0050  Luzhou Science and Technology Bureau — Grant Nos. 2024LZXNYDJ035 and 2020LZXNYDJ14  Cooperative Research Project of Deyang Second People’s Hospital and Southwest Medical University — Grant No. 2022DYEXNYD002  Special Project of Central Government Guiding Local Science and Technology Development of Sichuan Province — Grant No. 2024ZYD0269  The funders had no role in the study design, data collection, data analysis, interpretation of the findings, manuscript preparation, or the decision to submit the manuscript for publication.  Title Page (funding sources, grant numbers, and the role of funders). |
